# Job Satisfaction and Retention of Family Medicine Physicians in Primary Healthcare Centers in the Al-Qassim Region of Saudi Arabia: A Qualitative Study

**DOI:** 10.3390/healthcare14162578

**Published:** 2026-08-17

**Authors:** Ibrahim A. Aljabr, Abdulrahman A. Alharbi, Rehana Khalil, Moath Aljohani

**Affiliations:** 1 Department of Family Medicine, Armed Forces Hospital in Jazan, Ministry of Defense, Jazan 82714, Saudi Arabia; ibrahimaljabr1241@gmail.com; 2 Family Medicine Academy, Madinah 42313, Saudi Arabia; aaa.alharbi097@gmail.com; 3 Department of Family and Community Medicine, College of Medicine, Qassim University, Qassim 51452, Saudi Arabia; rn.noman@qu.edu.sa

**Keywords:** job satisfaction, physician retention, qualitative study, family medicine, Saudi Arabia, primary healthcare

## Abstract

**Background/Objectives**: Job satisfaction is important for high-quality healthcare delivery. This study aimed to explore family medicine physicians’ (FMPs’) opinions and beliefs regarding job satisfaction, factors influencing their satisfaction, and intentions to transition to other institutions. **Methods**: This qualitative descriptive study was conducted in the Al-Qassim region in 2024, using thematic analysis (Braun & Clarke) to analyze data. Eleven FMPs working within the health cluster of the Ministry of Health (MOH) were recruited using purposive sampling supplemented by the snowball technique. FMPs who were specialists and consultants were included. In-depth interviews were conducted, the interview content was transcribed, and thematic analysis was performed. **Results**: Four major themes were identified. The first theme was role ambiguity and workload burden; the second, resource gaps and operational limitations; the third, organizational barriers and professional stability; and the fourth, workplace dissatisfaction and intent to leave. Most of the FMPs reported being nearly satisfied with their job, and all agreed that dissatisfaction may lead physicians to consider leaving their institution. Job security within the MOH had a positive impact on job satisfaction, while concerns about changes associated with the health transformation process were noted. **Conclusions**: Physicians expressed varying degrees of satisfaction despite different challenges and limitations. Some physicians were open to changing their workplace, whereas one physician had already applied to other institutions. It is recommended to reassure physicians regarding job security during the health transformation process, clearly define job roles and responsibilities, and ensure the availability of necessary resources to improve physician satisfaction, provide better alignment with career expectations and maintain workforce stability in the MOH-affiliated PHC centers of the Al-Qassim region of Saudi Arabia.

## 1. Introduction

The sustainability of Primary Healthcare (PHC) is closely linked to the job satisfaction of Family Medicine Physicians (FMPs) [1]. Locke defined job satisfaction as “a pleasurable emotional state that arises from evaluating one’s job as achieving or facilitating the attainment of personal job values”, whereas job dissatisfaction reflects an “unpleasurable emotional state resulting from the perception that one’s job frustrates or blocks the achievement of those values or introduces disvalues” [2]. A study in England found that job dissatisfaction among FMPs was associated with increased intention to leave direct patient care. The intent to leave was ultimately a predictor of actually leaving direct patient care within 5 years; a higher proportion of physicians left patient care among those exhibiting higher intent [1]. A study conducted in Saudi Arabia found a negative association between FMPs’ satisfaction scores and their desire to change specialty, with each one-unit increase in satisfaction score associated with a 73% reduction in the likelihood of wanting to change specialty [3].

The Saudi Vision 2030 Health Sector Transformation Program (HSTP) positions primary healthcare as the foundation of its national healthcare system, shifting from hospital-centric care to a proactive, integrated model. Family physicians are central to this reform, managing population health through digital tools and value-based clinical pathways to deliver longitudinal preventive care within regional health clusters. It emphasizes the role of FMPs in improving public health and disease prevention and identifies physician attraction and retention as key challenges to achieving these goals [4]. In China, self-reported stressors related to job requirements or environment have been shown to be associated with turnover intentions among primary healthcare physicians, mediated by decreased job satisfaction [5]. Given this relationship, the importance of studying FMP job satisfaction and intentions to leave the institution becomes more evident in the context of healthcare transformation.

A systematic review of job satisfaction among nurse educators categorized the factors influencing job satisfaction into six levels: personal, organizational, managerial, academic, professional, and economic. Within these categories, high workloads, administrative burdens, inadequate resources, and limited organizational support emerged as key contributors to dissatisfaction [6]. In Saudi Arabia, key determinants of job satisfaction among PHC physicians in the Eastern region included the nature of work in primary care centers, as well as positive experiences with supervision, colleagues, and patient care [7]. Conversely, dissatisfaction is commonly associated with inadequate financial incentives, including low salaries and limited benefits [7,8], and with the perception that family medicine is less respected than other specialties [9]. Additional contributors include inadequate infrastructure and resources, such as laboratory, radiology, information technology, and support services [10]. Burnout related to high workload, patient pressure, paperwork, and poor organization has been consistently associated with lower job satisfaction across multiple regions of Saudi Arabia, including Jeddah, Al-Ahsa, and Tabuk [11,12,13].

Findings from Jeddah and the Eastern region of Saudi Arabia indicate that 62% of FMPs were satisfied [9]. In the Al-Qassim region, data showed a similar proportion of satisfaction, with 63.2% of primary care physicians reporting some or high satisfaction [14]. Most existing studies provide quantitative estimates but do not offer an in-depth understanding of the factors influencing satisfaction and dissatisfaction. To the best of our knowledge, no study in the Al-Qassim region has qualitatively examined job dissatisfaction and related factors among FMPs, particularly during the transformation process. This study aimed to explore FMPs’ opinions and beliefs regarding job satisfaction in PHC centers in the Al-Qassim region, the factors influencing their job satisfaction, and how these factors shape their intentions to leave their workplace.

## 2. Materials and Methods

### 2.1. Study Design and Setting

We utilized a qualitative descriptive research design to perform a comprehensive holistic analysis of job satisfaction among FMPs through open-ended questions and in-depth interviews with physicians. Data were gathered in 2024, and the geographical scope of the study included PHC centers operating under the Ministry of Health (MOH) health cluster in the Al-Qassim region of Saudi Arabia.

### 2.2. Sample Size and Technique

Participants were recruited primarily through purposive sampling, supplemented by snowball sampling. In the snowball sampling phase, initial participants referred eligible colleagues, facilitating access within a professional group not easily reached through institutional directories alone.

The final sample size was determined by data saturation. Data saturation was determined iteratively throughout the analytic process. The lead investigator assessed each new transcript for emerging codes and themes. Saturation was confirmed when successive interviews produced no new codes and the thematic structure remained stable, a judgment reached by discussion among the study investigators.

### 2.3. Inclusion and Exclusion Criteria

This study was limited to diploma- and board-certified FMPs (specialists and consultants) working in PHC centers in the Al-Qassim region. Interns or family physicians under training, general practitioners working as PHC physicians, physicians with other specialties working in PHC centers, and FMPs not engaged in clinical practice in PHC centers were excluded, as their contractual conditions, training status, and professional responsibilities differ substantially from those of qualified FMPs in terms of adequate clinical experience and decision-making responsibility in PHC settings, and including them would have introduced heterogeneity inconsistent with the study’s focused scope.

### 2.4. Data Collection

After obtaining ethical approval, physicians who met the inclusion criteria were contacted and provided with information about the purpose of the study. Upon agreeing to participate, the participants were sent a Google Form (Google LLC, Mountain View, CA, USA), which contained details on informed consent to participate in this study, ethical approval, time needed to complete the interview, and a questionnaire for demographic data and work characteristics. In-depth interviews with participants were conducted through an online platform (Google Meet, Google LLC, Mountain View, CA, USA).

The semi-structured interview guide (Supplementary Table S1) was adapted from factors affecting satisfaction found in the literature [15,16]. Additionally, the study investigators added some questions related to the local PHC context. As part of the interview guide, participants were asked to indicate their overall level of job satisfaction using a single-item Likert scale. This item was used to provide contextual information regarding participants’ overall satisfaction. Additionally, the interview guide was modified after piloting with FMPs from different regions who were not included in the final analysis, with the aim of assessing the study’s applicability, clarity, timing, and question flow. The following modifications were made after the pilot study. We expanded the interview duration from the initial 30 min to 60 min when coordinating with the interviewee, and we tested the question flow to ensure the interview progressed smoothly and to avoid guiding participants in a specific direction. The interviewer asked the necessary questions in the context of the interview, not in a fixed order. Additionally, we added a question regarding the ongoing health transformation, “Does the health transformation affect your job satisfaction?”, as it was concerning for physicians in the pilot study. The interview timing was selected by the participating physicians. To gain a clearer understanding of their perspectives, the participants answered in their preferred language, which was either Arabic or English (10 interviews in Arabic and 1 in English). The interviews were digitally recorded after obtaining consent from the physicians. Interviews conducted in Arabic were translated into English, with each interview lasting 30–60 min.

Each interview followed a consistent internal structure comprising two sequential components conducted within a single continuous session. The interview opened with free-attitude questioning, in which participants were invited to speak openly and without direction about their experiences of job satisfaction, and at the end of the interview, their intentions to remain in or transition away from direct patient care in PHC settings. This open approach provided a context for job satisfaction and reasons for retention or migration over time. It also helped to prevent “leading” individuals early on in their responses. Following the free-attitude opening, a semi-structured conversational approach was used within the same session to explore key topic areas more systematically. The conversational, affirming nature of this phase encouraged participants to elaborate on points raised during the free-attitude opening and to explore additional dimensions of their professional experience.

### 2.5. Transcription and Translation

All interviews were transcribed verbatim in Arabic or English. Transcripts were reviewed against the original audio recordings to verify accuracy. As the target publication language is English, Arabic transcripts were translated into English by a bilingual member [I.A.A.] of the research team, who works as a family medicine physician and is familiar with the local primary healthcare context. To minimize meaning loss during translation, the following strategies were applied: first, two bilingual team members [I.A.A. and M.A.] back-translated a selection of transcripts from English into Arabic, which were then compared with the original Arabic for semantic equivalence when needed; second, any discrepancies were resolved through discussion and consensus within the research team.

### 2.6. Data Analysis

Data were analyzed manually without qualitative data analysis software. Analysis followed the six-phase thematic analysis framework of Braun and Clarke (2006) [17], consistent with the study’s qualitative descriptive design. The phases were applied as follows: Phase 1—Familiarization: Translated transcripts were read and re-read in full by the lead investigator [I.A.A.], with initial observations recorded in a reflective journal maintained throughout the study. Phase 2—Generating initial codes: Transcripts were systematically coded inductively, with each code representing a discrete unit of meaning relevant to job satisfaction, influencing factors, or career intentions, recorded in a coding log by the lead investigator [I.A.A.]. As coding progressed, new codes were iteratively compared and refined against previously generated codes to ensure consistent application across the dataset. Phase 3—Searching for themes: Codes were reviewed, grouped by shared meaning into candidate themes, and a preliminary thematic tree was constructed manually to visualize relationships between codes, sub-themes, and candidate themes. Phase 4—Reviewing themes: the initial coding framework and candidate themes were reviewed by another investigator. The candidate themes were assessed for internal coherence and external distinctiveness against the coded extracts (Level 1) and full dataset (Level 2), with themes collapsed, refined, or discarded through iterative team discussion. The themes and subthemes were reviewed by all investigators, I.A.A., A.A.A., R.K., and M.A., until agreement was reached. Phase 5—Defining and naming themes: Each theme was formally defined, with scope and core meaning applied, and named to reflect its conceptual essence. Phase 6—Producing the report: Final themes were written up with supporting participant quotations, presented in English throughout the findings.

Categorical variables, such as demographic characteristics, are expressed as frequencies and percentages. Responses to the single-item Likert scale were summarized descriptively using frequencies and percentages; however, the primary analysis focused on the qualitative interview data.

### 2.7. Trustworthiness

Trustworthiness was established in alignment with Lincoln and Guba’s (1985) framework encompassing credibility, dependability, confirmability, and transferability [18]. Credibility was supported through engagement with the data via iterative re-reading and multi-phase coding, and peer debriefing through regular research team discussions in which analytic decisions were examined and challenged. The themes were developed from the original interview data using thematic analysis, and representative participant quotations are presented throughout the findings to illustrate and support the interpretation of each theme. Dependability was supported through maintenance of an audit trail documenting all analytic decisions, coding changes, theme revisions, and team discussions throughout the research process, an approach consistent with rigorous reporting practices demonstrated in comparable qualitative health research [19]. Confirmability was addressed through researcher reflexivity. The research team acknowledged their positionality as researchers with professional familiarity with the Saudi PHC context and maintained a reflexive journal throughout data collection and analysis to monitor how their own assumptions and professional experiences may have influenced interpretive decisions. These reflections were shared and examined within the team to minimize unexamined bias. Transferability was supported through provision of a detailed description of the study context, setting, sampling strategy, participant characteristics, and scope boundaries, enabling readers to assess the applicability of findings to comparable PHC settings or professional groups in similar institutional contexts. The authors acknowledge that findings reflect a specific institutional and regional context (MOH-affiliated PHC centers in Al-Qassim) and are not intended to be statistically generalized beyond this setting.

## 3. Results

We interviewed 11 FMPs (six men, 54.5%) who worked at different PHC centers (Table 1). All participants were board- or PhD-certified, and none had subspecialties in family medicine. Seven (63.6%) participants were classified by the Saudi Commission for Health Specialties (SCFHS) as consultants, and four (36.4%) were classified as specialist FMPs. Most participants (54.5%) had 2–5 years of experience, three (27.3%) had ≥10 years, one (9.1%) had 6–9 years of work experience, and one (9.1%) had ≤1 year. The number of clinics per physician varied during data collection according to the MOH health cluster schedule. During the study period, five participants (45.5%) worked 1–5 clinics per week, two (18.2%) worked 6–9 clinics per week, and four (36.4%) worked 10 clinics per week.

### 3.1. Themes and Subthemes

Four major themes were identified. Theme 1 included Role Ambiguity and Workload Burden, Theme 2 included Resource Gaps and Operational Limitations, Theme 3 included Organizational Barriers and Professional Stability, and Theme 4 included Workplace Dissatisfaction and Intent to Leave; these are presented in Table 2 with the number of physicians with the predominant view for each subtheme.

#### 3.1.1. Theme 1: Role Ambiguity and Workload Burden

- 3.1.1.a. Imprecise Duties and Professional Boundaries

The vague role definitions of specialist and consultant FMPs led to dissatisfaction and confusion. One physician stated,

“*Unfortunately, the institution I am affiliated with does not acknowledge the role of FMPs and is unable to distinguish between a general practitioner and a FMP. This perception is frequently expressed and can be discouraging, impacting various aspects such as the number of patients and clinic schedules, ultimately negatively affecting job satisfaction.*”

Other physicians agreed that vague job descriptions increased their dissatisfaction levels. This resulted in unclear distinctions between general practitioners, specialists, and consultants regarding the number of clinics per week. Most family physicians expressed dissatisfaction with the equalization of workloads, particularly when additional responsibilities, including administrative tasks, were assigned to those with higher classifications. This additional workload was perceived as discouraging, as they suggested that this dynamic could contribute to dissatisfaction among more experienced physicians.

- 3.1.1.b. Patient Overload and Time Constraints

Several physicians reported that patients often exceeded scheduled appointment limits, citing patient overload as a major concern. Officially, each patient is allotted 15 min, allowing for 16 appointments per clinic session and a total of 32 appointments per day. One physician commented on how this overload affected interactions with patients:

“*Unfortunately, my current role involves more service-oriented tasks than the application of family medicine principles due to patient volume and system constraints.*”

Conversely, another physician shared their satisfaction with scheduled appointments:

“*I believe that 15 min is sufficient and satisfactory for patient consultations, examinations, and education, provided that the PHC center continues to adhere strictly to scheduled appointments. Previously, the PHC center accommodated walk-in patients, with the number reaching up to 28–29 patients in a single clinic session, but now I am satisfied with the shift to scheduled appointments.*”

Family physicians shared their satisfaction with the current state of screening and preventive services, emphasizing both the progress and persistent challenges. One physician shared,

“*Screening programs for breast cancer and colon cancer have significantly advanced over the past 4 years. These national projects emphasize evidence-based practices and have positively impacted targeted populations.*”

This progress reflects the growing awareness and adoption of preventive measures in the healthcare system. However, the implementation of these screening programs has been described as time-consuming and sometimes overwhelming because of the additional workload placed on healthcare providers. Another physician noted,

“*Screening initiatives are beneficial for patients but add to our workload, as the processes are lengthy and require filling in papers (personal information, stamp, ID number).*”

Another physician added,

“*While I am responsible for performing screening tests in the clinic, I am also required as a physician to oversee one of these indicators and ensure the weekly target is met by remaining physicians, encouraging and motivating other doctors to perform the necessary screening. At the end of the week, I have to manually collect the cases into an Excel sheet and send it to the person responsible at the healthcare cluster.*”

FMPs affirm the need to offer flexibility in achieving these key performance indicators (KPIs) or assign a time slot to monitor such goals. Despite these challenges, the physicians expressed a strong belief in the value of these programs for improving public health outcomes. These findings suggest that frustration or dissatisfaction stems from the administrative burden of screening programs, rather than from the screening activities themselves.

- 3.1.1.c. Anticipated Burden of Urgent Care Responsibilities

All participants were currently working in day shifts. However, some had previous experience in urgent care clinics during their early career, where they covered night shifts. The following quotes reflect their anticipated concerns about working in evening/night shifts in an urgent care setting, based on these past experiences and hypothetical scenarios. FMPs reflected on the challenges associated with their previous work in urgent care clinics. One physician noted,

“*I have previously experienced working in urgent care during my early career, where I would cover shifts from 4 p.m. to 11 p.m. for an entire week. This was quite exhausting due to the unorganized patient flow, where it seems the main purpose was to manage as many patients as possible.*”

Most physicians expressed a marked preference for continuing in routine care rather than future involvement in urgent care responsibilities. One physician shared,

“*I do not prefer to work evening or night shifts, as I am inclined towards managing chronic illnesses rather than urgent or emergency cases.*”

Another added,

“*Most doctors enter the field of family medicine seeking stability. Although I can manage the situation, I will not be fully satisfied.*”

Another physician noted,

“*I prefer not to work in urgent care but would consider it if treated equally to emergency room physicians in regard to number of shifts.*”

Despite the challenges of working in urgent care clinics, one physician expressed a positive perspective, welcoming the experience and sharing,

“*I welcome the idea of urgent care; if I were assigned to it, it would not negatively affect my job satisfaction; however, if I were permanently assigned to urgent care, I would likely feel dissatisfied, as such a role could affect my lifestyle.*”

For some physicians, the anticipated demands of urgent care service were perceived as incompatible with the working conditions that currently support job satisfaction in routine PHC practice.

#### 3.1.2. Theme 2: Resource Gaps and Operational Limitations

Family physicians identified major challenges in accessing resources that are essential for providing optimum patient care.

- 3.1.2.a. Restricted Diagnostic and Laboratory Resources

Improvements in certain areas were acknowledged with the initiation of the Ayenati program (Supplementary Table S2) in PHC centers, including partial resolution of the challenges related to routine laboratory investigations, but challenges related to urgent laboratory and imaging investigations remain unsolved. Limited access to diagnostic resources was a recurring issue among participants. One physician explained,

“*The unavailability of radiological services at the PHC center negatively impacts job satisfaction. Access to imaging services such as X-rays, ultrasounds, computed tomography scans, and magnetic resonance images is crucial; personally, it enhances my knowledge by allowing me to review test results, and clinically, it enables me to better understand my patient’s health conditions.*”

Another shared,

“*Laboratory services are only available once a week, accommodating a limit of 20 patients (10 males and 10 females), and tests such as those for thyroid-stimulating hormone or erythrocyte sedimentation rates may require a wait of up to 2 weeks for the results.*”

Such delays hinder the timely and effective management of patients.

Physicians frequently cited the unavailability of essential laboratory resources necessary for urgent care, such as cardiac enzymes, complete blood count, urine dipstick, and other essential labs, as a significant limitation in urgent care clinics. One physician noted,

“*Tests or follow-up analyses cannot be performed in the evening. For example, cardiac enzyme tests are unavailable. If I performed an electrocardiogram (ECG) and it showed a normal finding, then I needed to conduct cardiac enzymes to rule out cardiac causes. How can urgent care operate without testing capabilities!*”

If these limitations recur across clinical encounters, they may become a persistent source of dissatisfaction that, over time, could weaken professional commitment and contribute to some physicians’ consideration of transitioning to better-resourced institutional settings.

- 3.1.2.b. Medication Availability

The majority of physicians noted a significant improvement in the availability of primary healthcare medications through the recently established Wasfaty system, but some identified a lack of readily available alternatives for certain conditions and inconsistent availability of medications in affiliated community pharmacies as ongoing concerns. Regarding prescription of medications, one physician noted,

“*There has been some improvement compared to previous times, but we still encounter limited options for alternative medication when indicated.*”

Additionally, the absence of pharmacists in PHC centers or through consultation is an emerging challenge that affects both physicians and patients. One physician stated,

“*The absence of a pharmacist in my practice makes it difficult to consult on medication availability and alternatives. Patients with chronic disease often return upset after visiting multiple pharmacies without finding their prescribed medications.*”

Such challenges put pressure on physicians when patients return to them complaining of an inability to obtain their prescribed medications. Participants reported that such situations contributed to frustration and reduced professional satisfaction, as they were unable to provide the level of care they considered appropriate.

- 3.1.2.c. Nursing Workforce Shortages and Training Gaps

Most physicians were satisfied with the current nursing staff’s competencies and adequacy; however, some highlighted areas for improvement, such as staffing shortages and gaps in training. One physician explained,

“*I work in an urgent care center, and in the laboratory, we need more nursing staff to draw samples faster. Currently, two nurses work with three doctors in the PHC centers. If I request an ECG for a patient and the nurse is busy or if the other doctor orders IV fluids for another patient, the remaining patients experience delays. Ideally, each doctor should have one or two dedicated nurses.*”

In addition to insufficient nursing staff, physicians highlighted the need for continuous training of healthcare providers in PHC centers. One physician shared,

“*Most of the nursing staff have older qualifications, typically holding diplomas, whereas bachelor’s degree holders and specialized nurses usually work in hospitals. I often have to instruct them on the correct way to measure blood pressure; for example: the patient should sit in a comfortable chair with their back supported for at least 5 min before the reading and the nurse should advise the patient not to talk while the measurement is being taken; despite these instructions, adherence is lacking.*”

Another noted,

“*The nursing staff are generally satisfactory and good at their work. However, they also need continuous training, particularly staff involved in clinical tasks.*”

For those who raised these concerns, these competency gaps increased workload due to a lack of teamwork and impacted care delivery. These challenges negatively affected their overall work experience and satisfaction.

- 3.1.2.d. Digital Platform and Clinical Workflow Challenges

Our participants shared their experiences with operational systems used in PHC settings (Raqeem, Mawid, Wasfaty, Ayenati, Sehaty, and Anat; see Supplementary Table S2), emphasizing the advantages and limitations. Physicians expressed satisfaction with the accessibility of electronic health records (EHRs, Raqeem), which facilitated continuity of care. One physician stated,

“*I find it satisfactory as it connects all PHC centers across the Kingdom.*”

However, concerns about fragmented systems remain as they are time-consuming and create inefficiencies in the workflow:

“*From my perspective, integrating these systems in one comprehensive platform, facilitating usability, and allowing for longer consultation time per patient can potentially enhance job satisfaction.*”

Physicians highlighted several user experience-related annoyances that hinder efficiency, including system slowdowns and frequent pop-ups. One physician explained that,

“*The Raqeem system is functional but could benefit from adjustments, such as reducing the repetitive pop-up screens that appear during patient file access, which can be quite frustrating.*”

In addition,

“*The Raqeem system is an improvement over previous systems; however, the issue of internet connectivity is concerning, as it can disconnect unexpectedly, prompting us to sometimes use our personal internet.*”

System reliability is a major concern, as unexpected shutdowns during clinic hours directly affect the ability of physicians to deliver timely care. One physician noted,

“*There are frequent updates, additions of features, or repairs made during working hours, which complicate our operations in the clinic and create issues with patients without prior notification. It is frustrating when the system malfunctions unexpectedly during clinic hours, affecting our workflow.*”

These challenges often force physicians to switch between digital and manual systems to manage their workload during system shutdowns, and when encountering these, no timely technical support is available:

“*In reality, when a problem occurs, it often takes a day to resolve, and even if you reach out to technical support, there is a delay in response.*”

Frequent technical issues and system limitations were perceived as obstacles to effective clinical practice, reducing professional satisfaction.

#### 3.1.3. Theme 3: Organizational Barriers and Professional Stability

Family physicians reported some challenges related to professional development and career stability. Although these factors do not directly affect clinical practice or the quality of care provided, they affect job satisfaction and decisions to remain in or change workplaces.

- 3.1.3.a. Inadequate Care Coordination and Referral Feedback

Family physicians reported notable challenges in integrating and communicating with hospitals and public health agencies. These challenges affect patient care, collaboration, and overall system efficiency. One physician explained,

“*Referring urgent patients requires a paper-based process, and I am unable to order laboratory tests or imaging directly.*”

Because EHRs at the PHC center (Raqeem) differ from those at the hospital, majority of physicians expressed dissatisfaction with the lack of effective feedback after referring patients to hospitals. One physician shared,

“*We do not receive feedback on referrals, nor do we have knowledge about what happens to our patients; communication between hospitals and PHC centers is inefficient.*”

These administrative deficiencies were perceived as contributing to job dissatisfaction and preference for more communicative and collaborative work environments.

- 3.1.3.b. Policy Instability and Regulatory Ambiguity

Most physicians expressed minimal effect of job policies on job satisfaction; however, inefficient communication arises as a source of increased dissatisfaction among FMPs. Regarding stability of roles and environment, one physician noted,

“*Frequent changes in policies at both PHC level and MOH or health cluster level create an unpredictable work environment.*”

Another physician noted, “*While the institution’s policies are fair, they are not well-known or consistently applied,*” which highlights the absence of clear insight about policies. The absence of clear communication fosters a culture of ambiguity and perceived organizational uncertainty. Others noted unofficial communication of new policies through WhatsApp. The frequent policy modifications, inconsistent implementation, and communication via informal channels may further contribute to psychological stress and reduced job satisfaction.

- 3.1.3.c. Constrained Career Growth

The lack of opportunities for skill enhancement and regular updates for physicians was a recurring issue. One physician stated,

“*I am highly dissatisfied with the lack of planned professional development. We need continuous training and workshops to review protocols. For example, if I need time off to attend a conference or workshop, or to learn a skill at a hospital, I am not granted permission, even if I offer to cover the expenses myself.*”

Another shared,

“*I had the opportunity to attend a training course in Jeddah 6 months ago, but my request for leave from my annual vacation balance was denied. There is no support for professional development, which negatively impacts my job satisfaction.*”

Even with the availability of professional development in hospitals, no effective notifications are provided to working physicians. One physician shared,

“*If there is a diabetes conference at the hospital, I do not receive notification and may only know about it by chance. Additionally, if there are delegation opportunities for attending conferences in other cities, I am not informed to get a chance to participate.*”

The physicians expressed that participation in educational responsibilities and engaging in discussion with trainees have a positive impact on keeping their clinical knowledge up to date. This was reflected in their dissatisfaction with the absence of educational tasks for some, as one physician shared,

“*I do not have educational responsibilities at this time, but I would welcome the opportunity to engage in them, as they would keep me updated on clinical developments and discussions.*”

This lack of structured professional development hinders physicians’ ability to stay up to date with advances in medicine. This may lead to the perception that the institution is insufficiently invested in their long-term professional career.

- 3.1.3.d. Incentives and Financial Compensation

The majority of participants reported being satisfied and having no income-related issues; a small number of specialist physicians raised income-related concerns that contributed to dissatisfaction with delayed promotion and financial allowances. One physician noted,

“*We are currently in the process of signing a new contract, and the issue is that I have been classified as a consultant by SCFHS for a year without any improvements to my situation in the workplace. With the new contracts, we have been informed that there will be no prospects for promotions for the next 2 years*.”

In addition, unclear overtime compensation policies further reduced satisfaction among family physicians. One physician stated,

“*My dissatisfaction arises from additional tasks assigned outside of working hours or targets that are unrelated to my core responsibilities. These additional duties cannot be completed within regular hours, and there is no compensation for them. This lack of reward is a significant factor contributing to my job dissatisfaction.*”

Another physician noted that the financial allowances, including those for training, have not been disbursed in a timely manner. Many physicians described unclear incentives and financial compensation such as reduced working days, and recognition for those working in urgent care clinics; this may be perceived not only as financial concerns but also as inadequate recognition of their contributions.

- 3.1.3.e. Perceived Job Insecurity

Many participants acknowledged that job security was a critical factor that influenced their decision to remain in their health cluster in the MOH. One physician shared,

“*The primary aspect contributing to my job satisfaction within the MOH sector is job security.*”

Other physicians agreed that job security is crucial for job satisfaction and working at the MOH. However, concerns about ongoing health transformation and its impact on job sustainability have also created anxiety. As one physician explained,

“*The transformation occurring within the MOH has created apprehension among employees regarding changes in work processes, salary structures, and job sustainability, with concerns about how performance will be assessed further contributing to this uncertainty, diminishing my overall sense of satisfaction.*”

Although most physicians were reluctant to express their views on the health transformation while waiting, uncertainty about their future careers created considerable anxiety among some of them.

#### 3.1.4. Theme 4: Workplace Dissatisfaction and Intent to Leave

- 3.1.4.a. Overall Satisfaction Spectrum and Perception

A single-item Likert-scale measure embedded within the interview guide was used to provide descriptive context regarding participants’ overall job satisfaction. The quantitative results presented below are descriptive only, whereas the other study findings are based on thematic analysis of the interview data. Satisfaction ratings indicated a generally positive but restrained pattern (Table 3). The majority of participants (54.5%) were nearly satisfied, while approximately one quarter (27.3%) expressed a neutral stance, and approximately one fifth (18.2%) were nearly dissatisfied. Notably, no participant reported either complete satisfaction or complete dissatisfaction. This suggests that while job satisfaction was more prevalent than dissatisfaction among FMPs in this setting, it was not absolute or unconditional, as it came with reservations rather than being complete and genuine fulfillment. These ratings provided quantitative context for the qualitative findings.

Importantly, a neutral satisfaction rating cannot be interpreted as an indication of contentment or stability within the participants. As the qualitative findings revealed, some of the participants who rated their satisfaction as neutral nonetheless expressed clear frustrations and gave active consideration to transitioning to other institutions. For instance, a physician who felt neutral about job satisfaction yet had applied to another institution explained,

“*I would rate my satisfaction as neutral. I feel that I am being compensated for work that lacks meaningful impact on society or the community. I believe my current role pays me more than the actual responsibilities I fulfill. Instead of engaging in clinical practice, diagnosing and treating patients, much of my work involves administrative tasks such as filling out paperwork, managing referrals, and refilling medications. These responsibilities could be handled by an intern. At this point, I am currently trying to change my job position to another institution, as my current institution has shown some hesitation in embracing improvement and maintaining reform efforts over time.*”

In contrast, another physician stated,

“*I believe I have not reached a level of job satisfaction, and the reasons for this include unattainable targets set within a specific timeframe mandated by higher authorities, but this would not lead me to leave and seek a position in another healthcare institution.*”

These accounts suggest that job satisfaction is closely linked to career expectations and that retention efforts should extend to physicians with neutral satisfaction, not just the overtly dissatisfied.

- 3.1.4.b. From Dissatisfaction to Intentions to Leave

All participants agreed that job dissatisfaction may lead some physicians to contemplate leaving their current institution. One physician stated,

“*If I find a position with more resources, that recognizes family physicians, with a satisfactory number of clinics, and with a comparable or higher salary, I would not hesitate to leave my current institution.*”

Another physician affirmed,

“*I can say that if my job satisfaction continues to decline, I will consider leaving my workplace. However, I am currently assessing my options as the distance from my home may not be a decisive factor if the current situation persists, considering that job satisfaction and workplace comfort take priority over the inconvenience of long distance from my home.” Additionally, one participant shared, “I am considering a move to a university hospital, particularly as a new family medicine department is being established, and I would welcome the opportunity to contribute to policy development in the department.*”

## 4. Discussion

In this qualitative study, we identified various factors affecting job satisfaction among physicians working in MOH-affiliated PHC centers in the Al-Qassim region of Saudi Arabia. These factors affected whether physicians were open to transitioning or applying to other institutions. Our findings showed that dissatisfaction may lead some physicians to consider leaving their institution, consistent with previous studies reporting that dissatisfaction among PHC physicians is associated with increased turnover intention [7,15,20,21].

Regarding structural, operational, and clinical work-related challenges, unclear job descriptions were a source of dissatisfaction; 8 out of the 11 FMPs working in PHC centers expressed dissatisfaction regarding imprecise duties and professional boundaries. Unclear roles and responsibilities negatively affect physicians’ job satisfaction by diminishing the perceived significance of their work and weakening their professional identity. These difficulties, together with challenges faced by health agencies in distinguishing between the responsibilities of general practitioners, specialists, and consultants, further contribute to uncertainty in workload distribution and administrative duties, and may reflect gaps in organizational support. Additionally, the feeling of limited community appreciation of FMP roles may contribute to reduced satisfaction, as shown in previous studies; more than half of respondents preferred to seek healthcare from other specialties than family medicine and did not clearly understand the FMP role [9,22,23]. During periods of health system reform, role clarity is particularly important for strengthening organizational commitment and physician retention. This is supported by a study conducted in a rural area in China following healthcare reforms, which found that certified physicians reported the highest satisfaction with the internal environment, job descriptions, and organizational management, highlighting the importance of clear roles and supportive organizational structures during healthcare transformation [24].

Furthermore, our study found that physicians anticipated future work in urgent care clinics would be associated with lower job satisfaction, a perception informed by their previous experiences in urgent care settings. Beyond the demands of urgent care itself, this finding may reflect concerns regarding work–life balance, professional well-being, and the sustainability of clinical practice, all of which are important determinants of physician retention and workforce stability [16,25]. However, because none of the participants currently worked in evening/night shifts or urgent care, these findings should be interpreted as anticipated attitudes rather than stable, routine experiences. Nevertheless, these anticipated concerns may be related to poor sleep quality and other health problems reported in the literature [26]. Additionally, a study of 176 PHC physicians in Switzerland found that satisfaction was lowest for working hours, suggesting that demanding schedules and extended working hours may negatively affect job satisfaction [27]. Collectively, these findings highlight the importance of organizational policies that support manageable workloads and work–life balance.

Our study showed clinical challenges such as ineffective resource allocation, including laboratory, imaging, medication, and nursing capacity gaps, which are crucial for managing patients and planning referrals or transitions to other institutions. These limitations contribute to dissatisfaction among physicians by restricting their ability to provide comprehensive and timely care. From a broader organizational perspective, inadequate resources may reflect gaps in institutional support and can undermine physicians’ confidence in the healthcare system’s ability to meet patient needs. This finding aligns with a study conducted in South Africa, which found that a lack of resources in the public sector frustrated more physicians than those working in the private sector [28]. In addition, family medicine trainers in Riyadh reported low satisfaction with the availability of resources, tools, and technology [29]. Together, these findings suggest that adequate resource allocation is not only essential for quality patient care but also for maintaining physician satisfaction, organizational commitment, and workforce retention.

Physicians interviewed after the implementation of the Wasfaty and Ayenati programs reported noticeable improvements in job satisfaction. However, opportunities for further improvement remain, including offering alternative medication options, expanding the Ayenati program to all PHC centers, and accommodating urgent laboratory requests in urgent care settings. This finding is consistent with a study that surveyed PHC physicians in Qassim province and reported generally favorable perceptions of Wasfaty, while also flagging a persistent request for a wider range of medication options within the formulary and continued connectivity issues that limit its benefit [30].

Patient-level satisfaction studies similarly show that while e-prescribing (Wasfaty) is broadly well received, satisfaction is consistently lower for medication availability than for other service dimensions in pharmacies [31,32]. These parallel findings from patient-level perspectives are consistent with the present study’s participants, who linked limited alternative medication options directly to frustration and a sense of reduced clinical autonomy. The participants’ recommendation to expand Ayenati to all PHC centers reflects barriers reported in primary care settings, where insufficient and variable availability of radiological and laboratory services has been identified [10].

A recurring concern among participants was the lack of structured feedback once a patient was referred from the PHC to secondary or tertiary care. This finding aligns with prior studies in Saudi Arabia, which similarly identified weak closed-loop communication between referring and receiving providers as a persistent limitation of the referral pathway [33,34]. Without feedback on diagnostic outcomes, treatment plans, or follow-up recommendations, family physicians in the present study described being unable to complete the therapeutic loop with their patients, which is linked to diminished continuity and coordination of care, which are core functions of family medicine as a specialty [35]. Integration and coordination have been perceived by healthcare professionals as among the barriers to healthcare transformation [36]. When referral feedback fails, the burden of care coordination falls back on the physician and the patient, often informally and without adequate clinical information. These findings indicate the need to account for these challenges during the transformation phase and afterward.

Participants frequently described a sense of unpredictability surrounding operational policies, staffing regulations, and role expectations. This pattern resonates with findings from a systematic review of job satisfaction among nurse educators [6] and with healthcare workers in public hospitals in Qatar [37], which found that satisfaction was negatively associated with role conflict and role ambiguity. The review suggests that unclear expectations and inconsistent role definitions may reduce job satisfaction regardless of workload or compensation, which offers a useful theoretical lens for interpreting the present findings. This alignment across different healthcare occupational contexts suggests that unclear role definition may be a determinant of job satisfaction across health professions more broadly, rather than a phenomenon specific to physicians or the Saudi context, and strengthens the argument that clearer, more consistently communicated policy frameworks could improve satisfaction levels.

Several participants expressed frustration at the limited pathways for professional advancement available to them within the PHC system. This finding is supported by the broader job satisfaction literature, as promotion opportunities were identified as a frequent determinant of job satisfaction across health professions in the Gulf region [38]. These promotion-related challenges may contribute to dissatisfaction and intention to leave.

Regarding non-clinical institutional challenges, our study showed that among participants who mentioned job security, it was acknowledged as a critical factor influencing their decision to remain in their health cluster within the MOH. This observation echoes the findings of a qualitative study in South Africa among physicians working in public and private hospitals; working in the public sector elicited satisfying and predictable working hours, job security, more structured interactive teamwork and academic environments, and income stability [28].

In the current study, we encountered some limitations, including those related to the study design itself, the lack of statistical inferences, limited hypothesis generation, and the highly interpretive nature of the qualitative study. Another inherent limitation of qualitative design, which limits the generalizability of the opinions shared during the interviews, is the small sample size. However, we continued data collection until saturation was reached to ensure that all relevant concepts had been captured. Additionally, we excluded interns or family physicians under training, general practitioners working as PHC physicians, physicians with other specialties working in PHC centers, and FMPs not engaged in clinical practice in PHC centers. This sampling strategy was intentionally chosen to ensure that all participants had substantial clinical experience and decision-making authority in PHC settings, which is directly relevant to the research question. As a result, our findings reflect the perspectives of a specific professional group of board- or PhD-certified family medicine physicians within MOH-affiliated PHC centers in a single region and may not be transferable to physicians with different qualifications, employment settings, or geographic contexts.

Moreover, limitations that may have contributed to variability in interviewees’ responses include ongoing changes in the national health sector and, more specifically, within individual health clusters. At the time of the interviews, the MOH health cluster had implemented a policy assigning a maximum of 10 clinics per week to both general practitioners and FMPs (specialists and consultants), which reduced distinctions in roles and responsibilities among these professionals. Some physicians had already experienced this change, whereas others had not. This discrepancy may have influenced participants’ responses regarding job satisfaction. Furthermore, none of the physicians were working in evening or night clinics at the time of the interviews, and all lacked current experience with urgent care shifts (evening and night). Consequently, their opinions about these settings reflect anticipated attitudes based on hypothetical scenarios and/or previous experiences, rather than current experiences in such environments. The results of this study are context-specific and do not represent situations across different institutions and regions in Saudi Arabia, as various levels of satisfaction and challenges are encountered among physicians therein, thereby hindering the generalizability of the findings. Furthermore, the reported number of participants’ views of each subtheme represents the predominant view from the analysis of qualitative interviews, not a prevalence distribution derived from quantitative questions; therefore, caution with interpretation is warranted.

However, our study provides insight into FMP opinions and beliefs in the Al-Qassim region and explores areas for potential improvement. By identifying the contributing factors to dissatisfaction, this study highlights actionable areas for improvement that could increase physician satisfaction, retention, and the quality of care, thereby improving PHC services in the Al-Qassim region.

### 4.1. Implications for Practice in MOH-Affiliated Primary Healthcare Centers in the Al-Qassim Region of Saudi Arabia

While the current study’s findings may require further investigation in quantitative research, the following are preliminary implications based on participants’ experiences and the themes identified in this study. These implications may help address the work-related and clinical challenges identified in MOH-affiliated primary healthcare centers in the Al-Qassim region of Saudi Arabia:

- Strengthen continuous medical education and training activities for physicians and nursing staff to support the knowledge and skills required for their roles.
- Consider expanding the Ayenati program to additional PHC centers and urgent care clinics.
- Improve employee support and communication by providing accessible informational materials outlining employees’ roles and responsibilities (e.g., job descriptions), supplemented by educational sessions. These measures would also help ensure that all personnel are fully informed and updated regarding ongoing changes.
- Improving the user experience in operational systems (Raqeem, Mawid, Wasfaty, Ayenati, Sehaty, and Anat) by integrating them into one platform, ensuring minimal system shutdown during working hours, and automating the extraction of KPIs (e.g., data on colorectal cancer screening services provided), thereby reducing physicians’ administrative workload. This approach may enable physicians to concentrate on their clinical work effectively.

### 4.2. Future Research

To gain a broader understanding of the factors influencing physician satisfaction and overcome the current study limitations, expanding qualitative research into other sectors, such as other governmental and private sectors, and into other regions of Saudi Arabia would allow for further in-depth exploration of the factors associated with physicians’ satisfaction. An additional quantitative study using a scale based on themes generated from previous and current studies would estimate the prevalence of these factors among family medicine physicians. Furthermore, longitudinal studies could be conducted to monitor how satisfaction changes over time rather than at a single point.

## 5. Conclusions

This qualitative study explored FMPs’ opinions and beliefs regarding job satisfaction in MOH-affiliated PHC centers in the Al-Qassim region of Saudi Arabia and their intentions to transition to other institutions. The findings revealed that job dissatisfaction can prompt FMPs to consider leaving or applying to other institutions. Reassuring physicians about their job security during the health transformation process, improving clarity of job roles and responsibilities, and improving and ensuring continuity of access to the resources needed for patient care in routine and urgent care settings may enhance satisfaction, align with career expectations, improve workforce stability, and reduce turnover intention in primary healthcare settings.

## Figures and Tables

**Table 1:** Sociodemographic data and work-related information on family medicine physicians (N = 11).

Variables		N	%
Sex	Male	6	54.5%
	Female	5	45.5%
Age	25–30	3	27.3%
	31–35	4	36.4%
	36–40	4	36.4%
Marital status	Single	2	18.2%
	Married	9	81.8%
Nationality	Saudi	10	90.9%
	Non-Saudi	1	9.1%
Level of education	PhD or Board	11	100%
	Fellowship	0	0%
Institution accredited by CBAHI	Yes	10	90.9%
	No	1	9.1%
SCFHS classification	Consultant Family Medicine	7	63.6%
	Specialist Family Medicine	4	36.4%
Years of experience	≤1	1	9.1%
	2–5	6	54.5%
	6–9	1	9.1%
	≥10	3	27.3%
Working time over the last month	Morning	11	100%
	Evening or Night	0	0%
Number of clinics per week *	1–5	5	45.5%
	6–9	2	18.2%
	10	4	36.4%

SCFHS, Saudi Commission for Health Specialties; CBAHI, Central Board for Accreditation of Healthcare Institutions. * Every 4 h was considered as one clinic.

**Table 2:** Themes and subthemes.

Theme	Subtheme	No. of Physicians with the Predominant View for Each Subtheme *
3.1.1. Theme 1: Role Ambiguity and Workload Burden	3.1.1.a. Imprecise Duties and Professional Boundaries	1 satisfied; 2 neutral; 8 not satisfied
	3.1.1.b. Patient Overload and Time Constraints	1 satisfied; 3 neutral; 7 not satisfied
	3.1.1.c. Anticipated Burden of Urgent Care Responsibilities	2 neutral; 9 not satisfied
3.1.2. Theme 2: Resource Gaps and Operational Limitations	3.1.2.a. Restricted Diagnostic and Laboratory Resources	1 satisfied; 8 not satisfied; 2 mixed opinion
	3.1.2.b. Medication Availability	9 satisfied; 1 not satisfied; 1 not addressed
	3.1.2.c. Nursing Workforce Shortages and Training Gaps	5 satisfied; 1 neutral; 3 not satisfied; 2 not addressed
	3.1.2.d. Digital Platform and Clinical Workflow Challenges	7 satisfied; 1 neutral; 3 not satisfied
3.1.3. Theme 3: Organizational Barriers and Professional Stability	3.1.3.a. Inadequate Care Coordination and Referral Feedback	5 satisfied; 6 not satisfied
	3.1.3.b. Policy Instability and Regulatory Ambiguity	3 satisfied; 5 neutral; 2 not satisfied; 1 mixed opinion
	3.1.3.c. Constrained Career Growth	2 satisfied; 4 neutral; 5 not satisfied
	3.1.3.d. Incentives and Financial Compensation	6 satisfied; 2 neutral; 1 not satisfied; 1 mixed opinion; 1 not addressed
	3.1.3.e. Perceived Job Insecurity	2 satisfied; 2 not satisfied; 1 mixed opinion; 6 not addressed
3.1.4. Theme 4: Workplace Dissatisfaction and Intent to Leave	3.1.4.a. Overall Satisfaction Spectrum and Perception	See Table 3
	3.1.4.b. From Dissatisfaction to Intentions to Leave	7 willing to leave; 2 intend to stay; 2 neutral

* Figures indicate the distribution of each view of each subtheme rather than a measured prevalence, given that these are results of interpretation of participants’ predominant view on the subtheme classified as satisfied, not satisfied, neutral, mixed opinion, or not addressed. Mixed denotes participants with both positive and negative views on the subtheme, e.g., in 3.1.2.a, satisfied with laboratory services but not with imaging. “Not addressed” denotes that participants did not discuss the subtheme because the interview was open-ended and participants were not asked about every topic.

**Table 3:** Distribution of job satisfaction ratings among family medicine physicians (N = 11).

Response	N	%
Satisfied	0	0.00
Nearly Satisfied	6	54.5
Subtotal: Satisfied	6	54.5
Neutral	3	27.3
Nearly Dissatisfied	2	18.2
Dissatisfied	0	0.00
Subtotal: Dissatisfied	2	18.2
Total	11	100

Note: Job satisfaction was assessed using a single five-point rating item administered within the semi-structured interview. Table 3 presents raw frequency counts and percentages of total participants.

## Data Availability

The datasets generated and analyzed during this study are not publicly available due to risks to individual privacy.

## Supplementary Materials

The following supporting information can be downloaded at: https://www.mdpi.com/article/10.3390/healthcare14162578/s1, Table S1: In-depth interview for job satisfaction for FMP; Table S2: Description of programs and systems.

## Abbreviations

The following abbreviations are used in this manuscript:

ECG	Electrocardiogram
EHR	Electronic health record
FMP	Family medicine physician
KPI	Key performance indicator
MOH	Ministry of Health
PHC	Primary healthcare
SCFHS	Saudi Commission for Health Specialties

## References

[1] Hann M., Reeves D., Sibbald B. (2011). Relationships Between Job Satisfaction, Intentions to Leave Family Practice and Actually Leaving Among Family Physicians in England. Eur. J. Public Health.

[2] Locke E.A. (1969). What Is Job Satisfaction?. Organ. Behav. Hum. Perform..

[3] Abdulrahman K.B., Alnosian M.Y., Alshamrani A.A., ALassaf H.I., Aldayel A.S., Alaskar Y.A., Alshehri M.A. (2021). Job Satisfaction Among Family Medicine Physicians in Saudi Arabia. J. Fam. Med. Prim. Care.

[4] Health Sector Transformation Program Delivery Plan. https://www.vision2030.gov.sa/media/u5xapka3/2021-2025-health-sector-transformation-program-delivery-plan-en.pdf.

[5] Ning L., Jia H., Gao S., Liu M., Xu J., Ge S., Li M., Yu X. (2023). The Mediating Role of Job Satisfaction and Presenteeism on the Relationship Between Job Stress and Turnover Intention Among Primary Health Care Workers. Int. J. Equity Health.

[6] Arian M., Soleimani M., Oghazian M.B. (2018). Job Satisfaction and the Factors Affecting Satisfaction in Nurse Educators: A Systematic Review. J. Prof. Nurs..

[7] Althumairi A.A., Bukhari F.M., Awary L.B., Aljabri D. (2023). The Effect of Transformation Policies on Healthcare Providers’ Satisfaction in Primary Healthcare Centers: The Case of Eastern Saudi Arabia. BMC Health Serv. Res..

[8] Allebdi A.A., Ibrahim H.M. (2020). Level and Determinants of Job Satisfaction Among Saudi Physicians Working in Primary Health-Care Facilities in Western Region, KSA. J. Fam. Med. Prim. Care.

[9] Bawakid K., Rashid O., Mandoura N., Shah H.U., Mugharbel K. (2018). Professional Satisfaction of Family Physicians Working in Primary Healthcare Centers: A Comparison of Two Saudi Regions. J. Fam. Med. Prim. Care.

[10] Mumenah S., Al-Raddadi R. (2015). Difficulties Faced by Family Physicians in Primary Health Care Centers in Jeddah, Saudi Arabia. J. Fam. Community Med..

[11] Bawakid K., Abdulrashid O., Mandoura N., Shah H.B.U., Ibrahim A., Akkad N.M., Mufti F. (2017). Burnout of Physicians Working in Primary Health Care Centers Under Ministry of Health Jeddah, Saudi Arabia. Cureus.

[12] Al-Haddad A., Al-Omar F., Al-Khaleel A., Al-Khalaf A. (2020). Prevalence of Burnout Syndrome and Its Related Risk Factors Among Physicians Working in Primary Health Care Centers of the Ministry of Health, Al Ahsa Region, Saudi Arabia, 2018–2019. J. Fam. Med. Prim. Care.

[13] Ishaq H.S., Ayub H., Albelawi S.H., Altawili M.A., Alenazi S.S., Omer H.M.I., Al Mamun M. (2025). Prevalence and Factors Associated with Personal and Work-Related Burnout Among Primary Health Care Physicians in Tabuk, Saudi Arabia. Saudi J. Health Syst. Res..

[14] Alhaqqas N.H., Sulaiman A.A. (2024). Job Satisfaction and Associated Factors Among Primary Healthcare Workers: A Cross-Sectional Study from Qassim Region, Saudi Arabia. Cureus.

[15] He R., Liu J., Zhang W.-H., Zhu B., Zhang N., Mao Y. (2020). Turnover Intention Among Primary Health Workers in China: A Systematic Review and Meta-Analysis. BMJ Open.

[16] Wen T., Zhang Y., Wang X., Tang G. (2018). Factors Influencing Turnover Intention Among Primary Care Doctors: A Cross-Sectional Study in Chongqing, China. Hum. Resour. Health.

[17] Braun V., Clarke V. (2006). Using Thematic Analysis in Psychology. Qual. Res. Psychol..

[18] Lincoln Y.S., Guba E.G. (1985). Naturalistic Inquiry.

[19] Nowell L.S., Norris J.M., White D.E., Moules N.J. (2017). Thematic Analysis: Striving to Meet the Trustworthiness Criteria. Int. J. Qual. Methods.

[20] Zhang Z., Shi G., Li L., Bian Y. (2020). Job Satisfaction Among Primary Care Physicians in Western China. BMC Fam. Pract..

[21] Maharani C., Afief D.F., Weber D., Marx M., Loukanova S. (2019). Primary Care Physicians’ Satisfaction after Health Care Reform: A Cross-Sectional Study from Two Cities in Central Java, Indonesia. BMC Health Serv. Res..

[22] Elagi A.A.A., Jaber B.A., Wassly A.H.A., Ahmed R.M.S., Bosily F.A.A. (2019). Public’s Perception and Satisfaction on the Role and Services Provided by Family Physicians in Saudi Arabia: A Cross-Sectional Study. J. Fam. Med. Prim. Care.

[23] Murad M.A., Kheimi R.M., Toras M.M., Alem R.H., Aljuaid A.M., Alobaidan J.N., Binishaq H.Y., Asiri A.A., Sagga M.K. (2022). Community Perspective on Family Medicine and Family Physician in Saudi Arabia 2020. BMC Prim. Care.

[24] Gu J., Zhen T., Song Y., Xu L. (2019). Job Satisfaction of Certified Primary Care Physicians in Rural Shandong Province, China: A Cross-Sectional Study. BMC Health Serv. Res..

[25] Steinbeck V., Ganguli I., Papanicolas I. (2026). Primary Care Physician Trends: Dissatisfaction, Stress, And Burnout In The US And 9 Comparator Countries, 2012–2022. Health Aff..

[26] Czyż-Szypenbejl K., Mędrzycka-Dąbrowska W. (2024). The Impact of Night Work on the Sleep and Health of Medical Staff—A Review of the Latest Scientific Reports. J. Clin. Med..

[27] Goetz K., Jossen M., Szecsenyi J., Rosemann T., Hahn K., Hess S. (2016). Job Satisfaction of Primary Care Physicians in Switzerland: An Observational Study. Fam. Pract..

[28] Ashmore J. (2013). ‘Going Private’: A Qualitative Comparison of Medical Specialists’ Job Satisfaction in the Public and Private Sectors of South Africa. Hum. Resour. Health.

[29] Al-Saab A.S., Barakat M., Alsaef A.M., Alnasyan A.Y., Altuwaijri M.M. (2022). Family Medicine Academy Trainers’ Satisfaction in King Saud Medical City, Riyadh, Saudi Arabia. J. Fam. Med. Prim. Care.

[30] Hammad M.A.M., Kalevaru C.S., Al Yahia O., Almutairi A., Almogbel F., Abalkhail B.M., Albahli R.A. (2023). Primary Health Care Physicians Perceptions About Centralized Electronic Prescription Service in Qassim Province, Saudi Arabia. Saudi J. Health Syst. Res..

[31] Almaghaslah D., Alsayari A., Almaghaslah S., Alsanna H. (2022). Patients’ Satisfaction with E-Prescribing (Wasfaty) in Saudi Arabia: A Survey of Country-Level Implementation. Healthcare.

[32] Alzahrani A.M., Alzhrani A.A., Felix H.C., Alharbi K.K., Shahzad M.W., Arbaein T.J., Monshi S.S. (2024). Patient Satisfaction with Private Community Pharmacies Versus Pharmacies in Primary Health Care Centers in Saudi Arabia. Saudi Pharm. J..

[33] Al Asmri M., Almalki M., Fitzgerald G., Clark M. (2020). The Public Health Care System and Primary Care Services in Saudi Arabia: A System in Transition. East. Mediterr. Health J..

[34] Alabbasi K.H., Kruger E., Tennant M. (2022). Strengthening Saudi Arabia’s Primary Health Care Through an e-Referral System: A Case Study. Clin. Pract..

[35] Ventres W.B., Stone L.A., Joslin T.A., Saultz J.W., Aldulaimi S., Gordon P.R., Lane J.C., Lee E.R., Prunuske J., Gildenblatt L. (2024). Storylines of Family Medicine III: Core Principles-Primary Care, Systems and Family. Fam. Med. Community Health.

[36] Al-Anezi F.M. (2025). Challenges of Healthcare Systems in Saudi Arabia to Delivering Vision 2030: An Empirical Study from Healthcare Workers Perspectives. J. Healthc. Leadersh..

[37] Yehya A., Sankaranarayanan A., Alkhal A., Alnoimi H., Almeer N., Khan A., Ghuloum S. (2020). Job Satisfaction and Stress Among Healthcare Workers in Public Hospitals in Qatar. Arch. Environ. Occup. Health.

[38] Alkhateeb M., Althabaiti K., Ahmed S., Lövestad S., Khan J. (2025). A Systematic Review of the Determinants of Job Satisfaction in Healthcare Workers in Health Facilities in Gulf Cooperation Council Countries. Glob. Health Action.

