# Supplementary material for: Job Satisfaction and Retention of Family Medicine Physicians in Primary Healthcare Centers in the Al-Qassim Region of Saudi Arabia: A Qualitative Study
Source: Healthcare (Basel). 2026 Aug 17;14(16):2578. doi: 10.3390/healthcare14162578 (PMC13511989; doi:10.3390/healthcare14162578)
Supplement: Supplementary file 1 [file healthcare-14-02578-s001.zip › Supplementary Table S1.pdf]

Supplementary Table S1. In-depth interview for job satisfaction for FMP.

<table>
<tr><td colspan="3">Do you feel comfortable speaking in English or Arabic throughout this interview?</td></tr>
<tr><td rowspan="2">General personal satisfaction</td><td colspan="2">What is your definition and idea of job satisfaction?</td></tr>
<tr><td colspan="2">Overall, how satisfied are you with your current role as a family physician in your facility (dissatisfied, nearly dissatisfied, neutral, nearly satisfied, or satisfied)? If not completely satisfied, what is missing for complete satisfaction?</td></tr>
<tr><td rowspan="3">Clinical-based satisfaction</td><td rowspan="3">How satisfied are you with the opportunity to apply evidence-based medicine (EBM) as a family physician at your institution?</td><td>Do you feel that you have an adequate time to build rapport, provide quality care, and practice preventive medicine for each patient?</td></tr>
<tr><td>How satisfied are you with the number of patients you see on a typical day? (Workload management)</td></tr>
<tr><td>How satisfied are you with access to (within your institution)
1. Necessary diagnostic tools (laboratory or test like spirometry or sleep study)?
2. Technology (for example imaging)?
3. Medication (even in Wasfaty)?</td></tr>
<tr><td rowspan="9">Institution satisfaction</td><td rowspan="9">What are the aspects of your institution that you find most satisfying? (Open question, at the end ask about specific aspect satisfaction): How satisfied are you with the</td><td>Salary?</td></tr>
<tr><td>Clinical information system (Raqeem or Mawid, Anat or others)? Internet connection?</td></tr>
<tr><td>Work in a shift system (in urgent care)?</td></tr>
<tr><td>Number of clinics per week?</td></tr>
<tr><td>Support from your manager at the PHC center level or higher?</td></tr>
<tr><td>Competency of health care workers (nurses or receptionists)?</td></tr>
<tr><td>Administrative task besides your clinical work, either at PHC or higher in health cluster?</td></tr>
<tr><td>Educational task besides your clinical work (in the training programs or in the clinic with trainees)?</td></tr>
<tr><td>Policy of your institution?</td></tr>
</table>

		Location of the institution (geographic area of work), and where do you work now?<br>1 Distance from your residence<br>2 Volume of patients<br>3 Management of the PHC center
		Promotion and development (availability of job number to whom eligible to be promoted)?
		PHC centers and main hospital collaboration<br>1 How satisfied are you with the level of collaboration between family physicians at PHC and specialists at your main hospital?<br>2 In family medicine practice, are there adequate referral pathways and available nearby appointments for patients requiring specialized care?<br>3 How satisfied are you with feedback about referred patients?
Does your satisfaction with your institution influence your decision to plan or change your institution? What is your best place to work in your opinion? (if satisfaction is low)		
What is the factor most definitely affecting your decision to stay or leave your institution?		
Is there any factor, in your opinion, that we did not discuss in this interview that you think will most likely affect physicians' job satisfaction?		
Does health transformation affect your job satisfaction? Explain?		
We have noticed that specialist and consultant physicians, after they finish training, try to decrease the number of clinics and perform administrative tasks instead. What do you think the reason for this?		
