# Supplementary material for: Job Satisfaction and Retention of Family Medicine Physicians in Primary Healthcare Centers in the Al-Qassim Region of Saudi Arabia: A Qualitative Study
Source: Healthcare (Basel). 2026 Aug 17;14(16):2578. doi: 10.3390/healthcare14162578 (PMC13511989; doi:10.3390/healthcare14162578)
Supplement: Supplementary file 1 [file healthcare-14-02578-s001.zip › Supplementary Table S2.pdf]

Supplementary Table S2. Description of Programs and Systems.

Raqeem	A comprehensive cloud-based Digital Health Record system, fully integrated and functioning as an EHR for PHC centers. Recently, it also included the referral and Wasfaty(e-prescription) systems.
Mawid	A Website that connects primary healthcare centers with hospitals and specialized centers to facilitate patient referrals from PHC to hospitals, specialized clinics, or services in other PHC centers, such as DEXA scans.
Wasfaty	An e-prescribing website intended to enable physicians in government primary healthcare centers and hospitals to issue electronic prescriptions, allowing patients to collect their prescriptions at private pharmacies affiliated to Wasfaty.
Ayenati	A new emerging program for laboratory technicians to coordinate physician-requested labs via the Rageem system from PHC centers, with sample transportation to hospital laboratories or centralized laboratories via Saudi Post Logistics. The result is then posted in Rageem.
Sehaty	A unified platform by the Ministry of Health that allows patients to receive sick leave certificates, medical reports, record vaccinations, and attend remote or virtual consultations.
Anat	A unified digital platform for health practitioners registered with the SCFHS. It supports health practitioners and service providers by enabling access to patient information to provide Telehealth or remote consultation services.

EHR, electronic health record; PHC, primary healthcare; DEXA, Dual-energy X-ray absorptiometry; FMP, family medicine physician; SCFHS, Saudi Commission for Health Specialties
